# Accurate Assessment of Moisture Content and Degree of Polymerization in Power Transformers via Dielectric Response Sensing

**DOI:** 10.3390/s23198236

**Published:** 2023-10-03

**Authors:** Anantawat Kunakorn, Sarawuth Pramualsingha, Peerawut Yutthagowith, Phethai Nimsanong, Supat Kittiratsatcha

**Affiliations:** 1 School of Engineering, King Mongkut’s Institute of Technology Ladkrabang, Bangkok 10520, Thailand; anantawat.ku@kmitl.ac.th (A.K.); sarawuth.pr@kmitl.ac.th (S.P.); supat.ki@kmitl.ac.th (S.K.); 2 Power System Analysis Section, Power System Control Department, Metropolitan Electricity Authority, Bangkok 10330, Thailand; phethai.ni@gmail.com

**Keywords:** degree of polymerization, dielectric response sensing, insulation condition assessment, k-nearest neighbor regression, oil-paper insulation, oil conductivity, moisture content

## Abstract

Power transformers are essential apparatuses used to transfer electrical energy from one voltage-level circuit to another. For reliable systems, preventive maintenance of the transformers is required to ensure good services of all mechanical, electrical, and insulation parts. Oil-immersed paper is most often used for transformer insulation. To ensure such good insulation performance and for assessing insulation conditions, advanced transformer sensing, monitoring, and effective assessment techniques are required. This paper introduces an effective technique for assessing the insulation conditions in power transformers, which are crucial for ensuring reliable energy transfer. The method utilizes advanced transformer sensing and monitoring, focusing on oil-immersed paper insulation commonly used in transformers. The technique employs dielectric response sensing, obtained from frequency-domain spectroscopy tests, to estimate degrees of polymerization (DP) and percentages of moisture content (PMCs) in the oil-immersed paper insulation. These parameters are well-known indicators of insulation performance. The approach is based on the weighted k-nearest neighbor regression, using a database of dielectric loss factors at low frequency and oil conductivities. To overcome limited data availability, linear interpolation and extrapolation techniques are applied to enlarge the database. Experimental verification and comparison with a previously developed method demonstrate the proposed technique’s superiority in accuracy and complexity. The maximum deviations of DP and PMC in the validation cases are 6.2% and 18.7%, respectively. In addition, to evaluate the validity of our proposed method in the case of a real power transformer, a comparative analysis of the DP and PMC values determined by the proposed method with those obtained through a previously developed and complicated approach was performed. The predicted results indicate that the DP and PMC values of the oil-immersed insulation fall within the ranges of 800 to 1000 and 1.5 to 2.0, respectively, which agree with the results determined by the complicated approach and closely align with real conditions. By offering a reliable and advanced means of assessing insulation conditions, this technique contributes to the preventive maintenance and overall efficiency of power transformers.

## 1. Introduction

Sustainability drives manufacturers to consider long-term environmental, social, and human impacts. Transformers, essential for electrical systems, can enhance sustainability through strategic design and maintenance modifications to reduce energy losses and environmental effects. Digitalization and condition-based lifecycle management [1] improve efficiency and extend transformer lifespan. Advanced maintenance approaches, based on accurate status data, optimize transformer maintenance, reducing risks and costs. Efficient strategies are crucial for asset management and prolonging transformer life.

Not only are advanced transformer sensing and monitoring required for transformer asset management, but also effective assessment techniques. Operators may optimize their maintenance planning and spending by keeping a close check on the most critical transformer components. According to CIGRE research [2], transformer monitoring with effective techniques can halve the danger of fatal failure. Early diagnosis of problems has been found to lower repair costs by 75% and revenue loss by 60%. Furthermore, annual cost reductions of up to 2% of a new transformer price can be realized, which results in significant savings for electricity utilities and companies.

Oil-immersed cellulose-based paper is utilized in various applications in the electrical and industrial sectors due to its unique characteristics, providing notable advantages for high-voltage apparatus insulation, including exceptional dielectric strength, elevated thermal stability, effective electrical insulation, resilience against environmental factors, and a long lifespan. Typically employed for insulating cellulose-based materials, mineral or transformer oil serves a dual role as a vital liquid insulation and a medium for heat dissipation in high-voltage equipment. However, with time, insulation degradation can transpire due to various factors. Historically, replacing used transformer oil was common, but this practice entailed environmental risks and financial burdens. Presently, transformer oil reclamation technologies introduce an efficient and sustainable solution. The reclamation process rejuvenates used oil, restoring its dielectric performance, prolonging its service life, and curbing toxic waste and emissions. This approach aligns with environmental responsibility and cost-effectiveness in high-voltage equipment maintenance. The reconditioning procedure mitigates degradation and acidic components in the oil, increasing oxidation stability, while diminishing gas solubility and water content. It effectively eliminates sediment from insulation and transformer windings, thereby reducing dissipation factor and losses, ultimately significantly elongating the transformer’s lifespan. Accurate data, encompassing dissolved gases, moisture content, and the degree of polymerization of cellulose-based paper, assume pivotal importance for the condition-based upkeep of power transformers.

The aging process of oil-immersed paper insulation refers to the gradual transformation that occurs in the insulation material over time due to a combination of environmental, thermal, chemical, and electrical stresses. The aging of oil-immersed paper is a complex and multifaceted phenomenon driven by various physical and chemical processes. When subjected to prolonged exposure to heat and oxygen, the cellulose molecules in the paper insulation undergo degradation. This degradation process results in the breakdown of cellulose molecules into smaller fragments and the release of functional groups, leading to the formation of polar groups. Simultaneously, the degree of polymerization decreases, causing the long polymer chains of cellulose to shorten. As aging progresses, it introduces impurities into both the paper insulation and the insulating oil. These impurities may originate from environmental contaminants or arise as breakdown byproducts from the insulation material itself. Aging also induces physical changes in the paper insulation, making it more porous. This increased porosity enables the paper to absorb moisture from the surrounding environment. Consequently, the moisture content within the paper rises, culminating in a higher dielectric loss factor. Furthermore, the aging process releases breakdown byproducts into the insulating oil, thereby contaminating it. Contaminated oil exhibits elevated electrical conductivity, which compromises its insulating properties. This elevated conductivity can increase the risk of electrical breakdown within the equipment, negatively impacting its performance and safety. Additionally, aging may release furanic compounds into the oil, further exacerbating the reduction in oil conductivity and contributing to an increase in the dielectric loss factor. These intricate processes collectively characterize the aging of oil-immersed paper, which can detrimentally affect the performance and longevity of electrical equipment.

The severity of the process depends on the level of thermal stress, oxidation, hydrolysis, chemical reactions, and electrical stress. This process predominantly affects the degradation of physical properties of the paper, i.e., loss in flexibility and change in dielectric constant, reduction in dielectric strength, increase in dielectric loss, and increase in conductivity of oil and paper insulation. Therefore, the degree of polymerization (DP) and percentage of moisture content (PMC) play important roles in the evaluation of the aging and remaining life of the oil-immersed paper insulation.

For preventive maintenance of high-voltage (HV) apparatuses including power transformers, it is well known that condition-based maintenance is more realistic, economical, and effective than time-based maintenance. Thus, it is necessary to appropriately assess the insulation conditions of the HV equipment for management of the condition-based maintenance [1,2,3,4,5]. The effective and crucial parameters correlating with the insulation conditions for the condition assessment of oil-immersed paper insulation are the percentage of moisture content (PMC) and the degree of polymerization (DP) because the PMC increases and the DP decreases during the aging process of the oil-immersed paper insulation [6]. It is recommended that the transformer insulation maintenance is performed when the PMC of the oil-immersed paper insulation is over 2%. When the DP becomes less than 800, the insulation is close to its life end. It is recommended to overhaul the transformer under this condition or replace it with a new one.

The DP tester based on viscosity testing and the moisture tester using Karl Fischer titration are utilized to determine the DP and PMC, respectively, of the test sample of oil-immersed paper insulation [7]. It is hard to retrieve the oil-immersed paper insulation from an actual transformer installed at the operation site for the determination of the DP and PMC. Therefore, some attempts have been made to investigate the relationship between the DP and PMC with other parameters that can be measured in practice. In [7,8] it was found that the DP and PMC have a very good relationship with the dielectric loss factor (loss tangent) and oil conductivity. In the study [9], an effective method for predicting the degree of polymerization (DP) and percentage of moisture content (PMC) was described. The technique involved integrating loss tangents across various frequency ranges and considering the oil conductivity. The loss tangent in the frequency domain was determined from frequency domain spectroscopy (FDS) results [10,11,12,13], while the oil conductivity was calculated based on known test cell dimensions, and the test sample conductance was derived from the flowing current and applied DC voltage [14]. However, it is important to note that the technique had a significant drawback, which is that it required a large amount of dielectric loss factor data across a wide frequency range, from 10^−3^ Hz to 10^3^ Hz. The development of the database involves time-consuming preparation of test sample materials and conducting of experiments. Therefore, a small database can be developed in practice. This aspect can pose challenges and inconveniences in practical applications.

To address the drawback in the previously developed technique [9], a new approach is introduced in this study, requiring only a small amount of input parameters and data for accurately assessing the moisture content and degree of polymerization in oil-immersed cellulose insulation via the sensing dielectric response. The proposed technique utilizes just two input parameters: the average loss tangent in the low-frequency range and the oil conductivity. To enhance the effectiveness of the model, the experimental database is expanded using linear interpolation and extrapolation techniques [15]. The proposed method also employs an adaptive k-nearest neighbor regression [16,17,18] for predicting the degree of polymerization (DP) and percentage of moisture content (PMC) in oil-immersed paper based on the low-frequency loss tangent and oil conductivity. Validation using other experimental data demonstrates the precision of the proposed model in evaluating the DP and PMC. Compared to the previously developed method [9], the proposed technique yields superior accuracy in predicting the DP and PMC. The proposed method offers an alternative approach explicitly tailored to assessing the average degree of polymerization (DP) and percentage of moisture content (PMC) across the entire oil-immersed insulation within high-voltage (HV) equipment. These averaged parameters serve as valuable indicators for evaluating the overall insulation conditions. Therefore, the proposed method stands as an attractive and effective approach for predicting the DP and PMC used for insulation assessment.

## 2. Development of the Proposed Method

As mentioned in the Introduction section, the fundamental concept underlying the proposed method for predicting the degree of polymerization (DP) and percentage of moisture content (PMC) is based on the aging process of the oil-immersed paper insulation. The development of the proposed method is composed of three parts, i.e., the selection of the input data in the prediction model, data preparation, and the prediction model based on an adaptive k-nearest neighbor regression. The descriptions of these parts of the proposed method are provided in this section.

### 2.1. Input Data Selection

**Figure 1:**
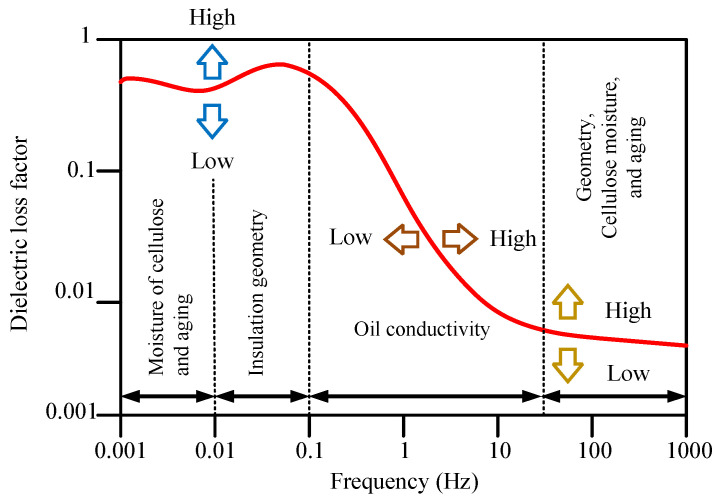
Loss tangent characteristics of the oil-immersed paper insulation.

**Figure 2:**
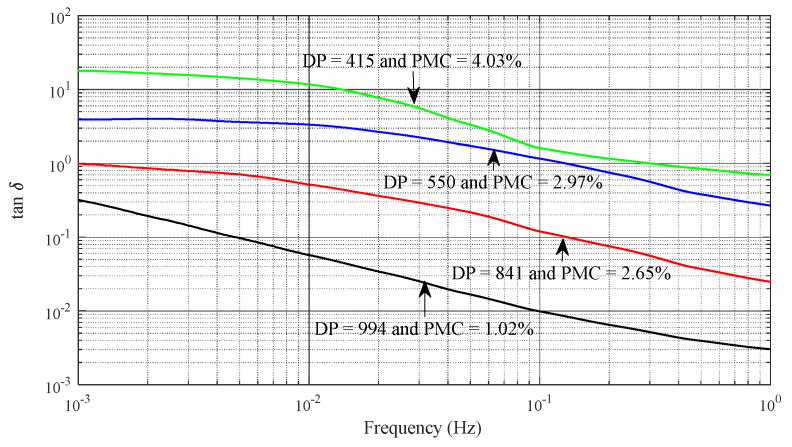
Loss tangent characteristics in the low frequency range of the oil−immersed paper insulation under different DPs and PMCs.

The prediction model for DP and PMC relies on insights from the aging process to guide the selection of crucial input parameters that must exhibit a robust correlation with DP and PMC. Similarly, the choice of input parameters for the estimating model, aimed at predicting DP and PMC in oil-impregnated paper insulation, draws upon well-established research findings. Specifically, it has been observed that both moisture and aging significantly influence the dielectric loss factor within the low-frequency range, as illustrated in Figure 1 [19]. Experimental studies conducted on samples of oil-immersed paper insulation with varying DPs and PMCs highlight that DP and PMC distinctly impact the loss tangent within the low-frequency range, as depicted in Figure 2. The combination of these observations reveals that as DP decreases and PMC increases, the loss tangent at low frequencies and oil conductivity intensify. Consequently, the chosen input parameters for the prediction model are the average loss tangent across the frequency range between 10^−3^ Hz and 10^−1^ Hz and oil conductivity.

**Figure 3:**
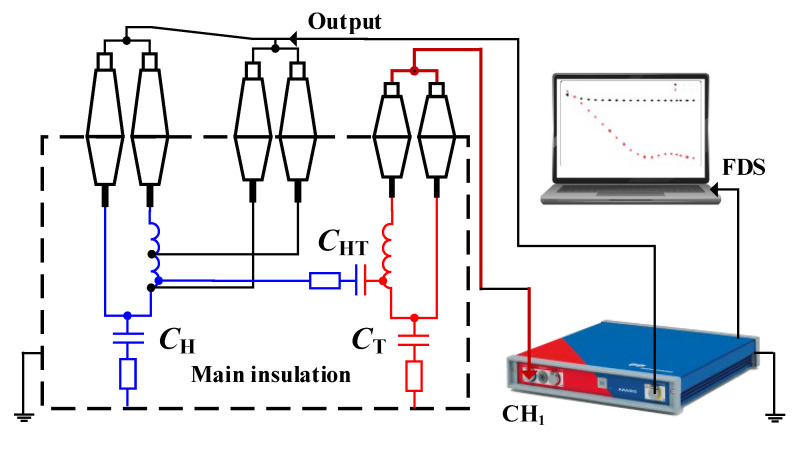
The equivalent circuit and test configuration schematic for the FDS test.

**Figure 4:**
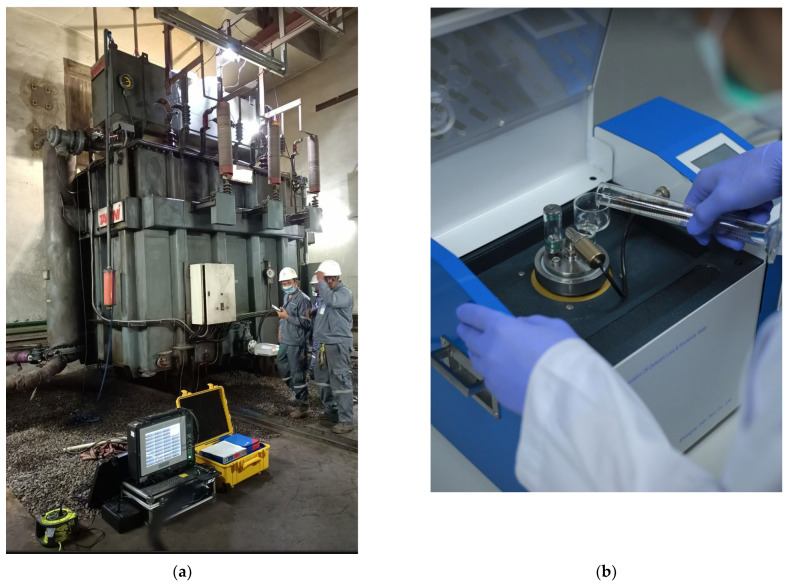
Experiments: (**a**) FDS test and (**b**) oil conductivity measurement.

The loss tangent is obtained through frequency domain spectroscopy (FDS), which measures complex capacitance and can be utilized to calculate the loss tangent by exposing the material to alternating electric fields across different frequencies. This reveals the material’s ability to store electrical energy under an electric field, offering insights into its behavior at various frequencies. The equivalent circuit and test configuration schematic for the FDS test on a power transformer are illustrated in Figure 3. Additionally, oil conductivity can be easily measured using the oil sample contained within a testing cell. Example photos of the FDS test and oil conductivity measurement are presented in Figure 4.

**Figure 5:**
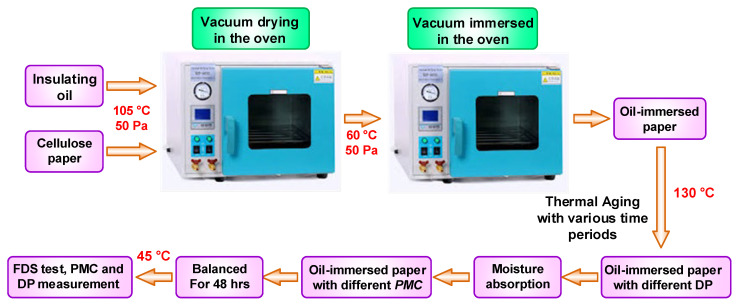
Experimental scheme for data development.

Some experiments on samples of oil-immersed paper insulations were performed by accelerating thermal aging with a temperature of 130 °C. Then the samples were exposed to the air to absorb the moisture, and the moisture was determined utilizing the gained weight, measured using a precision electronic scale. The oil temperature was carefully maintained at 30 °C during both the FDS test and the measurement of oil conductivity. This implies that the test temperature for the loss tangent curve of oil-immersed insulation within the oil remained at approximately 30 °C. The FDS tests of the samples were performed, oil conductivities were measured, loss tangents were calculated, and DP and PMC were measured. The above experimental scheme is illustrated in Figure 5.

### 2.2. Data Preparation

In practice, it is feasible to construct limited databases containing loss tangents and oil conductivities. However, certain prediction models demand substantial data to attain satisfactory accuracy, necessitating effective data normalization and enhancement techniques. In this paper, the k-nearest neighbor regression is applied for the prediction model because it has been recognized as essential for achieving such accuracy.

In this study, data from a total of 24 laboratory samples were gathered. We employed a straightforward validation set approach to assess the performance of our prediction model. Of these, the dataset of twenty laboratory cases was employed for the construction of the prediction model, and the corresponding results are detailed in Table 1. The remaining four samples were reserved for the validation of the proposed method.

**Figure 6:**
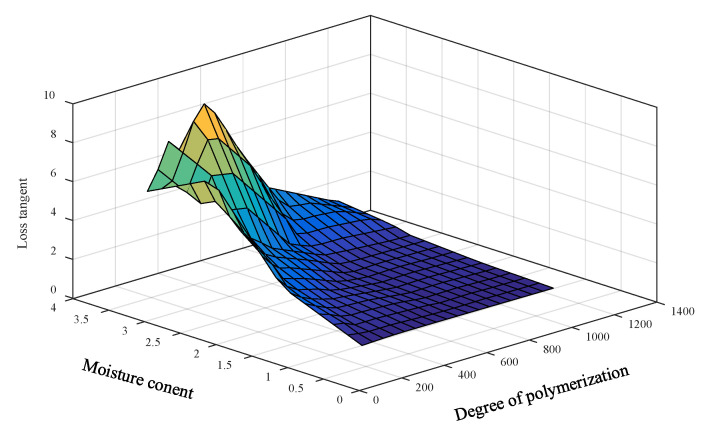
Relationship among the averaged low-frequency loss tangents, DPs, and PMCs.

**Figure 7:**
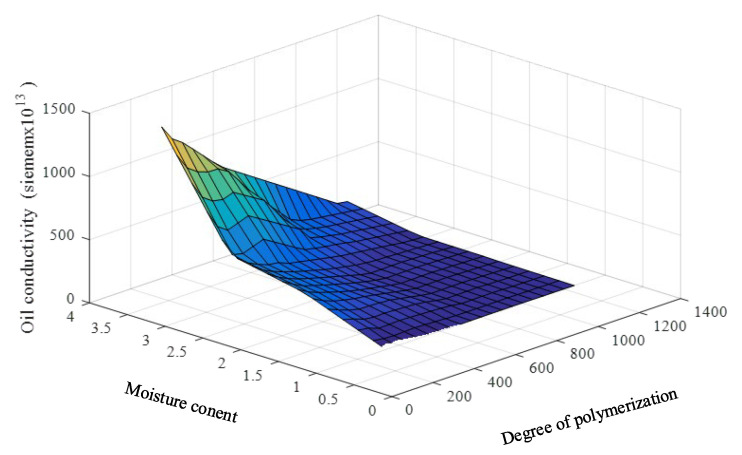
Relationship among the DP oil conductivities, DPs, and PMCs.

It is noticed that the loss tangent and oil conductivity in Table 1 tend to grow exponentially with a decrease in the DP and an increase in the PMC, as shown in Figure 6 and Figure 7. This is not a good characteristic for developing the prediction model because small changes in the DP and PMC have a large effect on the change in the loss tangent and oil conductivity. Moreover, linear interpolation and extrapolation are not appropriate for the data enlargement that is required in the model development.

**Figure 8:**
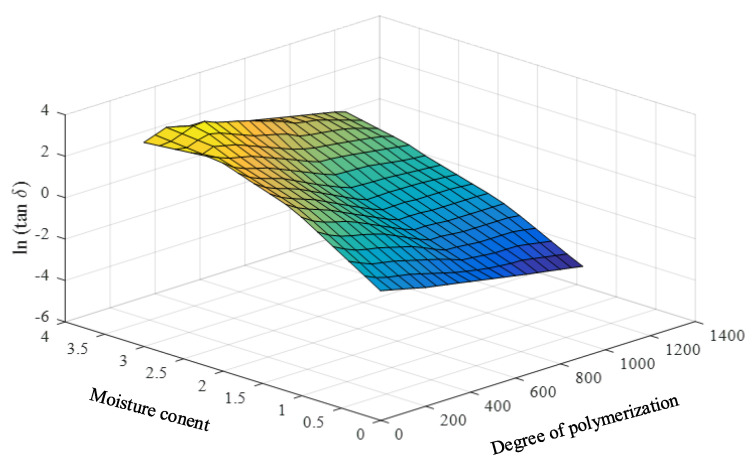
Relationship among the natural logarithms of the averaged low − frequency loss tangents, DPs, and PMCs.

**Figure 9:**
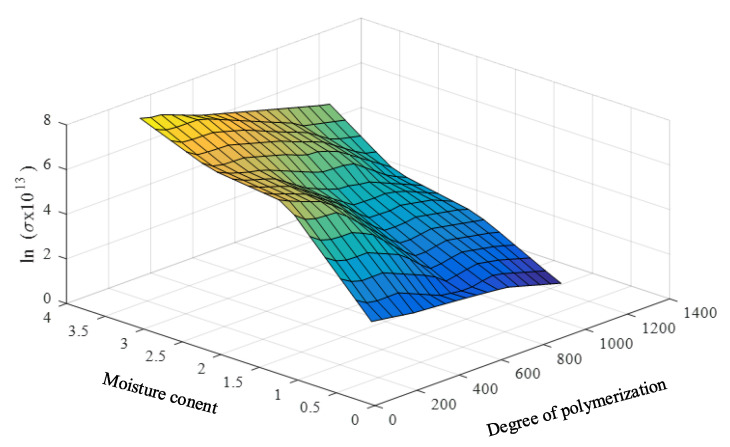
Relationship among the natural logarithms of DC oil conductivities, DPs, and PMCs.

To achieve practical and effective data enhancement, the linear relations of such parameters are required. Therefore, the natural logarithms of the loss tangents and the oil conductivities are utilized as the input parameters of the prediction model. The input parameters and the required and predicted parameters (DP and PMC) are found to have close linear relationships, as shown in Figure 8 and Figure 9.

The data preparation is composed of two processes. The first is data normalization. The input data are prepared by taking the natural logarithm of the low-frequency dielectric loss factors and the oil conductivities. Then, these input parameters are normalized by (1) to balance the effects of the different input data scales, where *x_i_*, *x_max_*, and *x_min_* are the considered, maximum, and minimum values of the input parameter, respectively, and *x_ni_* is the normalized parameter.

(1) $$x_{ni}=\left(\frac{x_{i}-x_{\min}}{x_{\max}-x_{\min}}\right).$$

In this paper, the k-nearest neighbor (kNN) regression method was applied to predict the DP and PMC. A weak point of this method is that it yields an average value in the vicinity of the analyzed data point. The output values for points situated near the dataset’s boundaries tend to exhibit pronounced deviations. To illustrate this, consider the test sample outlined in Table 2. Employing the same kNN regression technique, in the absence of data extension, the values for degree of polymerization (DP) and percentage of moisture content (PMC) amount to 744 and 1.66, respectively. Notably, these values deviate significantly, with the DP showing a −19.5% deviation and the PMC displaying a +31.5% deviation. To reduce the high deviation of over 30% at the point near the boundary, effective data extension is required. Therefore, the second aspect of this section involves data enhancement, which is a crucial step in addressing the practical challenge of limited dataset size and the boundary effect. To cope with the small amount of available data, both input and output data were extended. This extension was guided by the inherent trend observed in the prepared data, which displayed a near-linear relationship. To achieve this, a straightforward approach of linear interpolation and extrapolation was applied to the normalized data. In this paper, we offer a concise overview of linear interpolation and extrapolation techniques. These methods are well known and serve distinct purposes in data analysis, playing a vital role in bridging data gaps, estimating values, and projecting trends. Their utility extends across various domains, from engineering to economics.

Linear interpolation and extrapolation are foundational mathematical techniques with distinct roles in the realm of data analysis. Interpolation comes into play when the objective is to estimate values that fall between two well-established data points. It operates on the fundamental assumption that a linear relationship exists between these data points, meaning that the change in the dependent variable can be accurately predicted based on the changes in the independent variable within the known data range. Utilizing this assumption, interpolation calculates intermediate values, effectively connecting the dots along a straight line or linear curve. In contrast, extrapolation ventures beyond the known data points to predict values outside this established range. It extrapolates the linear trend identified within the provided data range, postulating that the same linear relationship persists beyond these bounds. While it also relies on the linear relationship assumption, extrapolation extends this assumption to make forecasts and estimations for values that lie beyond the observed data range.

**Figure 10:**
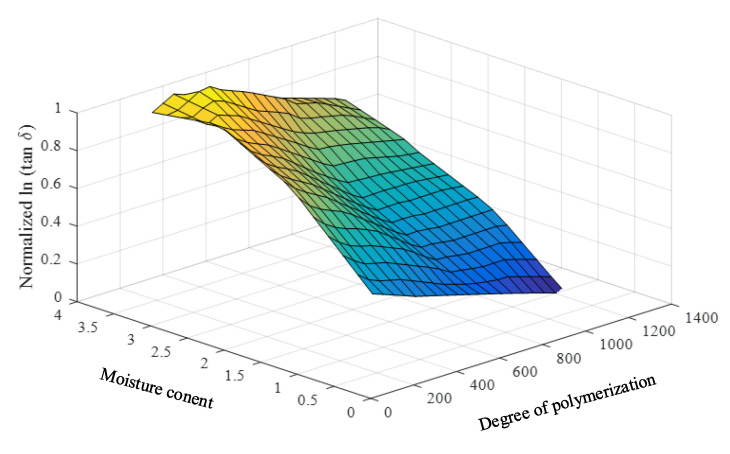
The normalized and enhanced data of the natural logarithms of the averaged low-frequency loss tangents, DPs, and PMCs.

**Figure 11:**
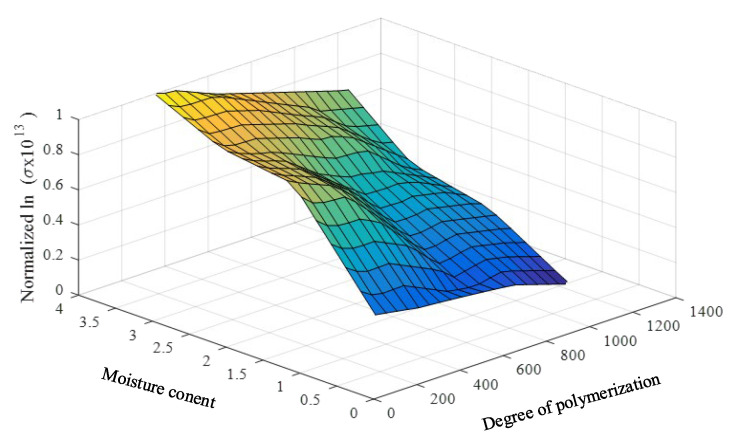
The normalized and enhanced data of the natural logarithms of DC oil conductivities, DPs, and PMCs.

From the original dataset (Table 1) size of 20, the data are enlarged to 304 cases in which the output parameters (DP and PMC) range from 1% to 4% (step of 0.2%) and 350 to 1250 (step of 50), respectively. The plots of the enlarged data are presented in Figure 10 and Figure 11.

### 2.3. Prediction Model

In the prediction model, the adaptive k-nearest neighbor (kNN) regression is applied to predict the DP and PMC using the enhanced database. A brief history and details of the kNN regression are presented in this subsection.

**Figure 12:**
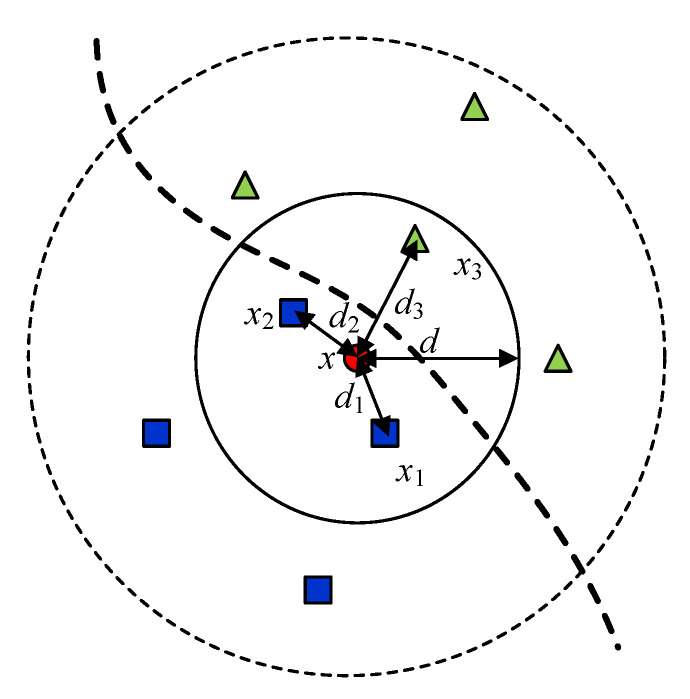
The considered data and data selection in the kNN algorithm with k = 3.

The kNN algorithm was invented by Evelyn Fix and Joseph Hodges in 1951 [16] and extended by Thomas Cover [17]. The algorithm can be used for classification and regression. In the classification kNN algorithm, the result obtained from the popular vote is a class of membership, and in the kNN regression, the result is an average value of the neighbors among the considered input data. Normalization of the data and weighting have been reported to significantly affect the accuracy of the method. Generally, the appropriate weighting approach and the number of k (the number of the considered data used for the result determination) are selected to obtain an accurate result. For example, the weight can be calculated from the inversed value of the distance (1/d) from the input to the selected neighbor. As shown in Figure 12, k is selected to be 3, and the data outside the solid line circle or the data having a distance over *d* are not considered in the prediction model. In the kNN classification, the predicted result (x) can be classified in the class of the blue square. In the kNN regression, the predicted result (x) is an average value in the specified area, and it can be calculated by (2).

(2) $$x=\frac{\sum_{i=1}^{k}\left(x_{i}/d_{i}\right)}{\sum_{i=1}^{k}\left(1/d_{i}\right)}$$

In this research endeavor, we employ an adaptive approach centered on the k-nearest neighbor (kNN) regression technique to predict two critical parameters: the degree of polymerization (DP) and the percentage of moisture content (PMC). Our prediction model is primarily driven by two input parameters, which are the average value derived from two fundamental attributes: the low-frequency loss tangent and the oil conductivity. A distinctive feature of our proposed method lies in its adaptive nature. Specifically, it entails a straightforward yet effective strategy for the selection of the parameter of k in the kNN algorithm. Instead of employing a fixed k value, our approach adaptively determines ‘k’ from the pool of data points with distances smaller than three times the minimum distance of the input data. This dynamic selection ensures that the nearest neighbors considered for prediction are not only close in proximity but are also highly relevant to the specific context.

**Figure 13:**
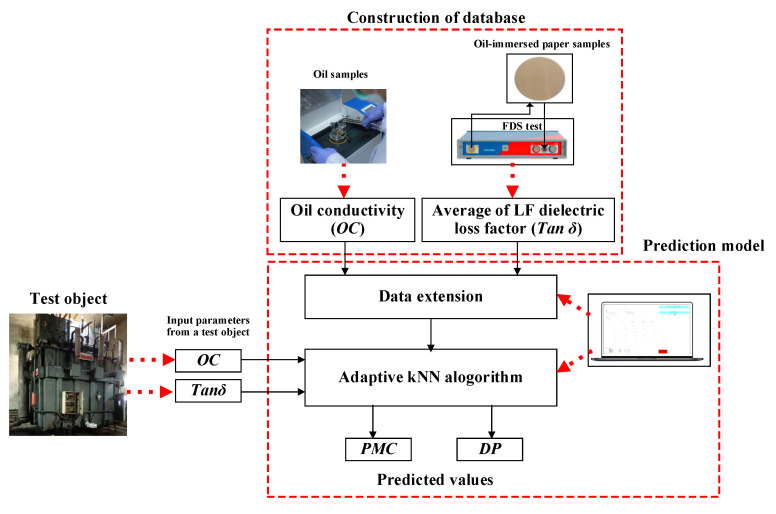
The visual schematic diagram of the proposed method.

Based on the description of the development of the prediction model, the visual representation of the model’s structure is presented in Figure 13. The input parameters consist of the low-frequency range loss tangent and oil conductivity, while the output parameters are DP and PMC. These outputs serve the purpose of assessing the condition of oil-immersed paper insulation and devising effective condition-based maintenance plans for high-voltage equipment utilizing such insulation. The efficacy of the proposed method is verified in the next section.

## 3. Verification of The Proposed Method

It is noticed that the proposed method can predict the DP and PMC of the enlarged database very precisely, because when the proposed method is employed to predict the parameters of the database, d equals zero, k equals one, and the results match perfectly with the parameters of the enlarged data. The proposed method is superior to the previously developed method [9] because the method uses only two parameters (the loss tangent in the low-frequency range and the oil conductivity), whereas the previously developed method uses four parameters (three integrals of loss tangent in the three frequency ranges from 10^−3^ to 10^3^ Hz and the oil conductivity).

To validate the proposed method, the remaining four sets of experimental data from the test samples (as detailed in Table 2) were utilized to predict the DP and PMC. A comparative analysis of the experimental and predicted results, involving both the previously developed and the proposed methods, is provided in Table 3. The findings indicate a strong agreement between the results predicted by the proposed method and the experimental data, with the proposed method demonstrating superior accuracy compared to the previously developed method, which is more complex and requires four input parameters.

Conducting measurements of the DP and PMC values on an operational transformer is rarely performed. Unfortunately, such data are unavailable to us. However, it is noteworthy that the selected transformer has been in operation for approximately 10 years. Given that the anticipated lifespan of a field transformer operating under normal conditions typically extends to around 40 years, this implies that the main insulation system of the transformer is presently in the intermediate phase of its operational life.

In the real case, the temperature during the measurement of FDS and oil conductivity may differ from the temperature of the test samples. However, it is possible to apply a temperature correction factor [9], as described in (3).

(3) $$\alpha_{T}=\frac{E_{a}}{R}\left(\frac{1}{T}-\frac{1}{T_{ref}^{}}\right)$$

where the activation energy is denoted *E_a_*, with a specific value of 113 kJ/mol, and the gas constant is represented by *R*, with a constant value of 8.314 J/(mol⋅K). *T_ref_* stands for the reference temperature of 318.15 K, while the test temperature is denoted *T*.

This correction factor can be directly multiplied with the average loss factor and oil conductivity [9] to convert the values from the reference temperature to the test temperature.

To evaluate the validity of our proposed method, a comparative analysis of the DP and PMC values determined by the proposed method with those obtained through a previously developed and complicated approach with the temperature correction factor [9] was performed. The predicted results presented in Table 4 reveal that the DP and PMC values of the oil-immersed insulation fall within the ranges of 800 to 1000 and 1.5 to 2.0, respectively, which agree with the results determined by the complicated approach [9] and closely align with real conditions. Encouragingly, we observed a substantial degree of concordance between the results. This suggests that, although not yet validated under actual transformer conditions, our proposed method holds promise and may be regarded as valid for providing approximate estimations.

## 4. Conclusions

In this research, a precise model was developed to predict the degree of polymerization (DP) and the percentage of moisture content (PMC) in oil-immersed paper insulation. Although substantial DP and moisture variations within transformers are observed in practical scenarios, the proposed method still offers an alternative approach that is explicitly tailored to assessing the average degree of polymerization (DP) and percentage of moisture content (PMC) across the entire oil-immersed insulation within high-voltage equipment. These averaged parameters serve as valuable indicators for evaluating the overall insulation conditions.

To enhance the available data, techniques involving linear interpolation and extrapolation were employed. The proposed approach utilizes an adaptive k-nearest neighbor regression, utilizing only two input parameters, namely the low-frequency loss tangent and oil conductivity, to predict DP and PMC. In this study, a straightforward validation set approach was employed to assess the performance of our prediction model.

The results indicate that the proposed method consistently aligns with the expanded dataset and corroborates other experimental findings. Notably, this method achieves these outcomes with the use of only two parameters, yet retains superior accuracy compared to the previously developed model, which relied on four parameters for prediction. The maximum deviations of DP and PMC in the validation cases are 6.2% and 18.7%, respectively. In addition, a comparative analysis of the DP and PMC values determined by the proposed method with those obtained through a previously developed and complicated approach was performed in the case of a real power transformer. The predicted results indicate that the DP and PMC values of the oil-immersed insulation agree with the results determined by the complicated approach and closely align with real conditions.

Based on these findings, it can be concluded that the proposed method presents an attractive approach for assessing the condition of oil-immersed paper insulation. Its ability to accurately predict DP and PMC with a reduced number of parameters enhances its practicality and usefulness.

## Figures and Tables

**Table 1:** DPs, PMCs, loss tangents, and oil conductivities of test samples.

Test Samples	DP	PMC (%)	Tan δ	σ (×10^−13^ S/m)
1	1285	1.10	0.018	1.13
2	1285	2.04	0.125	5.83
3	1285	3.12	0.386	11.70
4	1285	4.02	0.794	103.00
5	994	1.02	0.030	2.21
6	994	2.82	0.357	31.20
7	994	3.05	0.562	66.20
8	994	3.95	2.040	161.00
9	841	1.26	0.050	2.31
10	841	2.65	0.279	24.50
11	841	3.31	0.998	144.00
12	841	3.71	2.596	222.00
13	550	1.06	0.080	3.79
14	550	2.05	0.580	105.00
15	550	2.97	2.023	177.00
16	550	3.92	9.383	882.00
17	415	1.17	0.126	5.34
18	415	2.18	1.183	182.00
19	415	3.22	4.227	283.00
20	415	4.03	5.635	1070.00

**Table 2:** DPs, PMCs, loss tangents, and oil conductivities of test samples.

Test Samples	DP	PMC	Tan δ	σ (×10^−13^ S/m)
1	924	1.26	0.0494	6.5640
2	813	2.21	0.1816	47.100
3	541	3.07	2.4509	212.60
4	408	3.82	5.3061	792.65

**Table 3:** Comparison of the experimental and predicted results.

Experimental Results		Predicted Results and Errors from [9]		Predicted Results and Errors by the Proposed Method	
DP	PMC	DP	PMC	DP	PMC
924	1.26	1100 (+19.0%)	1.50 (+19.0%)	885.3 (−4.19%)	1.495 (+18.7%)
813	2.21	800 (−1.60%)	2.00 (−9.50%)	822.2 (+1.13%)	2.387 (+8.00%)
541	3.07	500 (−7.60%)	3.00 (−2.28%)	516.67 (−4.50%)	3.000 (−2.28%)
408	3.82	350 (−14.2%)	3.50 (−8.38%)	433.3 (+6.20%)	3.833 (+0.34%)

**Table 4:** Comparison of the predicted results.

Tan δ	σ	Predicted Results from [9]		Predicted Results by the Proposed Method	
		DP	PMC	DP	PMC
9.949	10.972	800	1.50	889.2	1.893

## Data Availability

Access to the data is restricted. The data were acquired from King Mongkut’s Institute of Technology Ladkrabang and can be obtained through the corresponding author with King Mongkut’s Institute of Technology Ladkrabang’s approval.
